# Novel requirements for HAP2-mediated gamete fusion in *Tetrahymena*

**DOI:** 10.21203/rs.3.rs-2928984/v1

**Published:** 2023-05-15

**Authors:** J.F. Pinello, J. Loidl, E.S. Seltzer, D. Cassidy-Hanley, D. Kolbin, A. Abdelatif, F.A. Rey, R. An, N.J. Newberger, Y. Bisharyan, H. Papoyan, H.M. Byun, H.C. Aguilar, E. Cole, T.G. Clark

**Affiliations:** 1 Department of Cell Biology and Molecular Genetics, University of Maryland, College Park, MD, 20742 USA.; 2 Department of Chromosome Biology, Max Perutz Labs, University of Vienna, Vienna, Austria.; 3 Department of Microbiology and Immunology, Cornell University, Ithaca, NY, 14850 USA.; 4 Unité de Virologie Structurale, Institut Pasteur, 75724 Paris, France.; 5 CNRS UMR 3569, 75724 Paris, France.; 6 Office of Technology Development, Harvard University, Cambridge, MA, 02138 USA.; 7 Biology Department, St. Olaf College, Northfield, MN, 55057 USA.; 8 These authors contributed equally to the work.

**Keywords:** HAP2, membrane fusion, *Tetrahymena*, ZFR1, membrane protrusion

## Abstract

The ancestral gamete fusion protein, HAP2, catalyzes sperm-egg fusion in a broad range of taxa dating to the last eukaryotic common ancestor. Remarkably, HAP2 orthologs are structurally related to the class II fusogens of modern-day viruses, and recent studies make clear that these proteins utilize similar mechanisms to achieve membrane merger. To identify factors that may regulate HAP2 activity, we screened mutants of the ciliate *Tetrahymena thermophila* for behaviors that mimic *Δhap2* knockout phenotypes in this species. Using this approach, we identified two new genes, *GFU1* and *GFU2*, whose products are necessary for the formation of membrane pores during fertilization and show that the product of a third gene, namely *ZFR1*, may be involved in pore maintenance and/or expansion. Finally, we propose a model that explains cooperativity between the fusion machinery on apposed membranes of mating cells and accounts for successful fertilization in *T. thermophila’s* multiple mating type system.

## Introduction.

The conserved transmembrane protein, HAP2 (also known as GCS1^1^), drives gamete fusion in a vast number of species^2,3^. At the same time, HAP2 orthologs are structurally similar to viral class II fusion proteins^4–6^ and catalyze membrane merger by mechanisms that mimic those used by Dengue, Zika, and related viruses for host cell entry. This begins with the activation of HAP2 protomers at the plasma membrane of one cell (typically, the male gamete) leading to insertion of hydrophobic “fusion loops” into the lipid bilayer of the partner cell (usually the female gamete)^7–11^. Activation is followed by trimerization of HAP2 ectodomains and conformational fold-back to deform membranes and bring them close enough to allow them fuse^8–10,12^.

While dynamic rearrangements in HAP2 structure generate the forces necessary for gamete merger, mechanistic details around the activation of pre-fusion protomers, the possible transition of hemi-fusion intermediates to full fusion pores, and the expansion of pores to form a single contiguous membrane are only just beginning to emerge. These steps regulate both cell-cell and virus-host cell fusion in other contexts^13–15^ and may be critically important for the precise spatiotemporal control of fertilization.

In flowering plants and green algae, timely activation of the HAP2 machinery appears to be regulated, in part, by accessory proteins that sequester the fusogen and/or constrain it in a prefusion conformation prior to membrane merger. In the unicellular alga, *Chlamydomonas reinhardtii*, the lineage-specific adhesion protein, MAR1, associates either directly or indirectly with HAP2 in minus (“male”) gametes and is required for its correct expression and localization on a single, apical membrane protrusion known as the *minus* mating structure^16,17^. Interaction of MAR1 with the broadly conserved GEX2 family member, FUS1, on *plus* (“female”) gametes releases HAP2 from an inactive state allowing it to drive gamete fusion at the site where mating structures of *plus* and *minus* gametes adhere^9,16^.

A somewhat analogous situation has been described in the model plant species, *Arabidopsis thaliana*, where HAP2 is initially sequestered on intracellular vesicles of male gametes through associations with the plant-specific proteins, DMP8/9, until sperm and egg come into close proximity^18^. Under the influence of EC1, a secreted peptide from the egg, HAP2 translocates to the plasma membrane of sperm where it promotes gamete fusion^19^. Whether additional proteins are involved in the regulation of fusion pore opening and/or expansion in plant or algal systems is unknown.

Along with *Chlamydomonas* and *Arabidopsis*, the free-living ciliate, *Tetrahymena thermophila* offers a powerful model for the study of membrane dynamics during fertilization^20^. Following starvation, cells undergo a program of sexual development that generates up to seven different mating types^21,22^. A given mating type can recognize and adhere to any of the other six mating types but not its own. Fertilization can be readily synchronized and begins at a specialized region of membrane at the apical ends of two mating cells known as the conjugation junction where large numbers of HAP2-mediated fusion pores open up^23–25^. Pore expansion allows reciprocal exchange of migratory haploid pronuclei across the junction culminating in karyogamy and the development of new progeny that come apart following membrane repair^24,26^ (Fig. S1).

Unlike sexually dichotomous species where the protein is expressed primarily in male gametes^17,27,28^, HAP2 is produced by all seven mating types of *T. thermophila* and is required in both cells of a mating pair for efficient pore formation to occur^4,25^. While complementary mating types lacking *HAP2* can recognize and adhere to one another, the absence of the fusion protein from one cell of a mating pair results in a steep decline in the number of pairs capable of forming fusion pores, while its absence from both cells completely abrogates pore formation^4^. In the absence of fusion pores, mating pairs fail to exchange meiotic pronuclei and, when left undisturbed, separate prematurely without generating progeny^25^. Furthermore, compared to wild-type cells, pairs formed by *Δhap2* deletion strains come apart readily when agitated suggesting that junctional pores help to “rivet” these highly motile cells together long enough to allow them to complete fertilization^25,29^.

Based on these findings, it seemed reasonable that unstable pairing between mutant mating types might offer a useful screen to identify additional gene products involved in membrane pore formation during fertilization. Using this approach, we identified two hypothetical genes designated *GFU1* and *GFU2* (Gamete FUsion 1 and 2) that are necessary for HAP2-mediated pore formation in mating *T. thermophila*. In addition, we show that a third gene, referred to as *ZFR1*^30^, while necessary for pair stability, is not required for pore opening. The implications of these findings are discussed in terms of possible mechanistic roles for GFU1, GFU2, and ZFR1. Finally, direct observations of *HAP2* mutant cell lines provide strong evidence of cooperative interactions between the fusion machinery on apposed cells. A model that explains cooperativity and accounts for the expression of HAP2 in all seven mating types of *T. thermophila* is presented.

## Results

### Identification of genes required for stable pairing of mating cells.

Sexual development in *T. thermophila* is initiated by nutrient deprivation^20^. Following starvation, physical interactions between cells of different mating types lead to rapid upregulation of genes involved in cell-cell recognition and adherence, membrane fusion, meiosis, and exchange of migratory pronuclei between mating cells resulting in karyogamy^20–22,31^. Initially, interactions between cells are transient, but within 1.5 hr post-mixing (30°C) complementary mating types begin to form mechanically stable pairs that remain adherent until they naturally separate as sexual progeny roughly 12 hr after their initial interaction (Fig. S1).

In separate studies to identify genes involved in meiosis^32^, we found two instances in which deletion of a hypothetical gene that is upregulated during conjugation (TTHERM_000161578 and TTHERM_00569470 [Fig. S2A,B]) resulted in unstable pairing between complementary mating types lacking the same gene. In each case, mutant strains recognized and adhered to one another at the conjugation junction (Fig. 1A-D) but could be separated by vigorous pipetting. As demonstrated here, we find that both genes are required for cells to form fusion pores and name them *GFU1* and *GFU2*, respectively.

To better characterize pair formation by *Δgfu1* and *Δgfu2* deletion strains, we compared the kinetics of pairing and the relative strength of cell-cell adhesion in crosses between knockout (*Δgfu* × *Δgfu*), wild type (WT × WT), and WT × *Δgfu* cell lines. When left undisturbed, complementary mating types carrying the *Δgfu1* deletion reached the same degree of pairing with roughly the same kinetics as WT × WT cultures over the first 6 hr of mating (Fig. 1E). Similarly, mixtures of *Δgfu2* deletion strains reached nearly WT levels of pairing (albeit with slower kinetics) over the same time frame (Fig. 1F). In both cases, however, the pairs formed between complementary *Δgfu1* × *Δgfu1* or *Δgfu2* × *Δgfu2* deletion strains prematurely exited the sexual cycle and came apart several hours before WT × WT pairs (Fig. 1E,F). Notably, *Δgfu1* and *Δgfu2* deletion strains behaved differently from each other when mated to WT cells. As shown in Figure 1, pairs formed by WT × *Δgfu1* cells had an intermediate phenotype in which many, but not all pairs, came apart prematurely before 12 hr (Fig. 1E), while WT × *Δgfu2* cells showed essentially the same kinetics of pairing and cell separation as WT × WT crosses (Fig. 1F).

To measure the relative strength of cell-cell adhesion, mating pairs were vortexed at a fixed speed and examined microscopically for pair stability at varying intervals. As expected, WT × WT pairs were highly stable, whereas a majority of pairs formed between *Δgfu1* × *Δgfu1* (Fig. 1G) or *Δgfu2* × *Δgfu2* (Fig. 1H) deletion strains came apart relatively quickly after vortexing. When mixed with WT cells, *Δgfu1* and *Δgfu2* deletion strains again behaved differently from each other. Whereas WT × *Δgfu1* pairs were unstable, WT × *Δgfu2* pairs were indistinguishable from WT pairs in resisting mechanical shear (Fig. 1G,H). Taken together, these results indicate that GFU1 (like HAP2) is required in both cells of a mating pair for stable pairing to occur, whereas GFU2 confers pair stability when expressed in only one cell of a mating pair.

### Requirements for GFU1 and GFU2 in membrane fusion.

To determine whether pair instability was correlated with an inability of *Δgfu1* or *Δgfu2* deletion strains to form fusion pores, we used a previously described flow cytometry assay to measure exchange of labeled cytosolic proteins between mating cells as a proxy for membrane pore formation during fertilization^4^. In these assays, starved cells of different mating types were labeled either red or green with CellTrace^™^ Far Red (CTFR) or carboxyfluorescein succinimidyl ester (CFSE), respectively, then combined and fixed either immediately after mixing or 18–24 hr post-mixing after mating pairs had separated as postzygotic progeny (Fig. 2A,B).

Figure 2 shows representative flow cytometry plots of mating cultures containing 1:1 mixtures of WT × WT, WT × *Δgfu*, or *Δgfu* × *Δgfu* strains. When fixed immediately after mixing (0 hr), two intensely labeled cell populations were always present, one bright green (CFSE^hi^) and the other bright red (CTFR^hi^) representing the labeled cells of different mating types before membrane fusion (Fig. 2C). In WT × WT cultures, following fusion and pair separation, these initial populations were almost entirely replaced by two new populations of labeled cells, one more green than red and the other more red than green (Fig. 2D). These new populations (delineated the “Mid” gate in Fig. 2D), represent single cells that had paired, formed fusion pores, and exchanged cytosolic dyes before separating as newly formed progeny. Additionally, a small population of CFSE^hi^/CTFR^hi^ cells was almost always present (delineated the “Pairs” gate in Fig. 2D) representing cells that continued to pair even at late time points^4^.

Unlike WT × WT crosses, matings between *Δgfu1* × *Δgfu1* and *Δgfu2* × *Δgfu2* deletion strains showed little evidence of dye exchange with the percentage of cells in the “Mid” gates of flow cytometry plots averaging <1% at 18–24 hr post-mixing (Fig. 2E,F,I). Thus, despite being able to form pairs, these cells, like *Δhap2* deletion strains, were unable to form fusion pores at the conjugation junction.

In the case of WT × *Δgfu* crosses, data from flow cytometry fusion assays were consistent with the pair stability results described above. Only a small percentage (5–15%) of cells were capable of fusion in WT X*Δgfu1* crosses (Fig. 2G,I), whereas in WT × *Δgfu2* crosses the percentage of cells capable of fusion was essentially the same as for WT × WT matings (Fig. 2H,I). These results reinforce the idea that pore formation and pair stability are closely linked and demonstrate a bilateral versus a unilateral requirement for the *GFU1* and *GFU2* gene products, respectively, for efficient membrane fusion between mating pairs.

### Possible Functional Roles of GFU1 and GFU2.

At the level of primary sequence, GFU1 is predicted to be a 557 amino acid protein with no obvious transmembrane domains (Fig. S2E). GFU2, on the other hand, is almost certainly a transmembrane protein with a predicted N-terminal signal peptide (residues 1–17), a 146 amino acid ectodomain, a single transmembrane helix (residues 164–186), and a 42 amino acid C-terminal cytosolic region that is enriched in positively charged amino acids (estimated pI = 11.46) (Fig. S2F).

Using BLAST alignment tools, no homologs outside the ciliate taxon were detected for either GFU1 or GFU2 at the level of primary amino acid sequence. To gain insight into their potential functions we used AlphaFold2^33^, along with other protein structure modelling algorithms to deduce 3-dimensional structures for both proteins. Based on this analysis, GFU1 was predicted to be almost entirely alpha-helical with its long antiparallel hairpins bearing a close resemblance to F-BAR (Fes/CIP4 homology-Bin/Amphiphysin/Rys) domains on proteins that bind and regulate curved membranes^34,35^ (Fig. 3A,B). In the case of GFU2, other than the transmembrane helix, weak structural homologies with other proteins were confined to a region containing three sets of anti-parallel β-sheets within the ectodomain (Fig. 3C). These similarities were not considered to be functionally significant.

To determine the intracellular location of GFU1, we replaced the endogenous gene with chimeric constructs encoding either mCherry- or HA-epitope tagged versions of GFU1 at their C-termini. In both instances, transgenic cell lines paired normally with WT cells and with complementary mating types expressing the same GFU1 tagged proteins (Fig. 3D-F)). Furthermore, consistent with its requirement in membrane fusion, GFU1 chimera localized along the entire length of the conjugation junction punctuated by small gaps (Fig. 3D,E). When GFU1:mCherry was co-expressed with an inducible, C-terminal GFP-tagged version of HAP2, the two proteins showed overlapping and non-overlapping patterns of staining (Fig. 3F). To date, we have been unable to generate tagged versions of GFU2 due to the high AT-content of its corresponding cDNA.

HAP2 orthologs from *Arabidopsis* and *Tetrahymena* have been shown to promote low levels of cell-cell fusion when expressed as recombinant proteins in cultured mammalian cells^4,6^. To determine whether we could use this approach to study potential interactions between GFU1, GFU2, and HAP2, we undertook efforts to measure syncytia formation in cultures of BSR-T7 baby hamster kidney (BHK) cells following expression of the plant or ciliate fusion proteins either with or without co-expression of both GFU1 and GFU2. When expressed alone, HAP2 orthologs from both species drove modest levels of cell-cell fusion compared to a known viral class I fusion protein from Nipah virus^36^, with *Arabidopsis* HAP2 showing roughly twice the level of activity as the *Tetrahymena* protein. Interestingly, co-expression of GFU1 and GFU2 with *Tetrahymena* HAP2 resulted in a nearly 2-fold increase in activity while no effect was seen with the *Arabidopsis* HAP2 ortholog suggesting that species-specific interactions between ciliate proteins may occur (Fig. S3).

### Cooperativity between the fusion machinery on apposed membranes.

While the interplay between HAP2 and the newly identified GFU1 and GFU2 proteins remains unclear, both GFU1 and HAP2 are required in both cells of a mating pair for efficient pore formation to occur (Fig. 2I). This raises the interesting possibility that the fusion machinery on apposed membranes of mating cells somehow interacts, an argument bolstered by the fact that the 75–85% decline in fusion efficiency observed when HAP2 is absent from one cell of a mating pair cannot be compensated for by massive overexpression of the native fusion protein in the wild-type partner^4,20^.

To address this question further, we decided to revisit the fusogenic capacity of cells carrying a mutation to a highly conserved arginine residue (R^164^), which likely stabilizes the HAP2 fusion loop(s) through formation of a salt bridge with an equally conserved glutamic acid residue (E^108^) present in virtually all HAP2 orthologs^5^. In previous studies using *Tetrahymena*, substitution of an alanine residue for the conserved arginine at position 164 had no effect on the ability of mutant cells to fuse with WT strains^4^. This was surprising since the same mutation abrogates the fusogenic capacity of HAP2 in species where the protein is present on only one gamete membrane^5,8^.

To determine whether R^164^A mutant protein was fully functional or whether the WT protein on the apposed membrane was able to spare an otherwise defective gene product, we examined the ability of *R*^*164*^*A* × *R*^*164*^*A* strains to form fusion pores using flow cytometry. As in previous studies, reciprocal crosses between WT cells and cells carrying the mutant allele showed robust fusion (Fig. 4A,C). However, pairs formed between *R*^*164*^*A* mutant cell lines were completely unable to fuse (Fig. 4B,C). These results clearly support the involvement of R^164^ in the formation of a loop-stabilizing salt bridge and establish the importance of the loop structure for HAP2-mediated fusion of *Tetrahymena* mating types. More importantly, the ability of the WT protein to rescue the *R*^*164*^*A* mutation provides further evidence of cooperativity between the fusion machinery on apposed membranes of mating cells.

At least one possible model to explain such cooperativity invokes trans-interactions between HAP2 ectodomains on either side of the conjugation junction (see below, Discussion)^6,20^. Nevertheless, while such interactions are theoretically possible, GFU1 (which also has a bilateral requirement for efficient fusion) is almost certainly not a transmembrane protein making homotypic (trans) interactions across the junctional interface unlikely. Still, additional evidence of cooperativity involving GFU1 came to light in efforts to validate the functionality of HA- and mCherry-tagged versions of the protein.

As shown in Fig. 4, cells expressing HA-tagged GFU1 showed WT levels of fusion when mated to each other (Fig. 4D,H). By contrast, cells expressing the mCherry-tagged version underwent very low levels of fusion (<3% of cells) indicating that the larger tag renders GFU1 essentially non-functional (Fig. 4E,H). However, when crossed to WT cells, transgenic cells expressing the GFU1:mCherry chimera underwent roughly 50% fusion, substantially greater than that seen in crosses between WT cells and *Δgfu1* deletion strains (Fig. 4F-H). The ability of native GFU1 in WT cells to restore the activity of a defective GFU1:mCherry chimera in their mating partners, along with its inability to compensate for loss of the protein in deletion strains, would suggest that cooperative interactions between GFU1, or between protein complexes/structures containing GFU1, are somehow required for membrane fusion.

### ZFR1 in pair stability and fusion.

Previously, Xu et al. found that ZFR1, a zinc-finger protein that localizes specifically to the conjugation junction is required for *T. thermophila* mating types to form mechanically stable pairs^30^. Structure prediction algorithms suggest that ZFR1 may be an E3 ubiquitin ligase (Fig. S4), and as such, could alter the half-lives of proteins involved in stable pairing including those required for membrane fusion. Given the link between pair stability and membrane pore formation described here, we predicted that cells lacking *ZFR1* would be unable to fuse. To test this, we deleted the *ZFR1* coding sequence in mating types carrying different drug resistance markers in their gametic micronuclei and examined the characteristics of mating, as well as overall fertility, in crosses between *Δzfr1* × *Δzfr1*, WT × *Δzfr1*, and WT × WT strains.

When left undisturbed, complementary mating types lacking ZFR1 recognized one another and formed pairs with similar kinetics and slightly reduced efficiency compared to WT × WT and WT × *Δzfr1* cultures (Fig. 5A). Nevertheless, when mechanically agitated, *Δzfr1 × Δzfr1* pairs behaved more like *Δhap2* knockout pairs, coming apart within seconds of vortexing essentially as described by Xu et al.^30^ (Fig. 5B). To determine whether these cells were able to fuse, mating types were labelled with fluorescent dyes and subjected to flow cytometry assays as described above. Consistent with their ability to form mechanically stable pairs, crosses between WT and *Δzfr1* strains showed nearly wild type levels of fusion (Fig. 5C,E). Surprisingly however, *Δzfr1* × *Δzfr1* knockout pairs also showed high levels of fusion despite their unstable pairing phenotype (Fig. 5D,E).

We then examined the ability of *Δzfr1* × *Δzfr1* knockout pairs to generate progeny by crossing parental cell lines carrying either cycloheximide or 6-methylpurine drug resistance markers in their germline micronuclei, isolating individual pairs, and determining the drug resistance phenotypes of resulting synclones after refeeding (Table S1). Consistent with the findings of Xu et al.^30^, we saw that only 22.5% of mating pairs gave rise to true progeny (compared to >80% in standard crosses between WT cells^25^). In short, while a majority of *Δzfr1* deletion pairs were able to generate fusion pores, a relatively small percentage gave rise to progeny. As argued below, alterations to pore size and/or the kinetics or pore opening could affect the exchange of meiotic pronuclei across the junctional interface between cells and lead to reduced fertility.

## Discussion.

As shown here, *Tetrahymena* mating types carrying deletions in either *GFU1* or *GFU2* were able to recognize and adhere to their homologous deletion strains, but, as in the case of *Δhap2* knockouts, were unable to form fusion pores. The corresponding GFU1 and GFU2 gene products can therefore be added to a list of lineage-specific factors, that together with MAR1 in *Chlamydomonas reinhardtii*^16^, and the DMP8/9 family members of *Arabidopsis thaliana*^18,37,38^, appear to regulate HAP2-mediated gamete merger during fertilization.

From a functional standpoint, MAR1 and the DMP8/9 family members play critical roles in the localization and timely activation of HAP2 in algae and plants, respectively^16,18,37,38^. Whether the same is true of the newly identified GFU1 and GFU2 proteins is unclear. GFU1 differs markedly from the algal and plant proteins based on its predicted structure and lacks obvious transmembrane domains. GFU2, on the other hand, is a single-pass transmembrane protein that could potentially interact with receptors on the apposed membranes of mating cells. Indeed, given the reduced kinetics of pairing in crosses between complementary *Δgfu2* deletion strains (Fig. 1F), GFU2 could act as an adhesion protein, and like MAR1^16^, participate in activation of the HAP2 fusion machinery as a member of a hypothetical receptor-ligand pair or signaling complex.

That said, it is unlikely that GFU2 interacts directly with HAP2 as appears be the case for DMP8/9^18^, and possibly MAR1^16^ as well. If such interactions occur, one would predict that the absence of GFU2 in one mating type would have a significant impact on HAP2-mediated fusion in *T. thermophila*, which is clearly not the case (Fig. 2H,I). However, the ability of GFU2 to act unilaterally from a single membrane does raise an interesting parallel with another protein involved in cell-cell fusion, namely, Myomerger (Minion/Myomixer)^15,39^. In concert with Myomaker, a multi-pass membrane protein required on adjacent cells, Myomerger has been shown to resolve hemi-fusion membrane pore intermediates generated by Myomaker during syncytia formation in vertebrate striated muscle^15^. GFU2 and Myomerger are both small, singlepass transmembrane proteins, and while they differ at the level of primary structure, it is not inconceivable that they are functionally related. Experiments are currently underway to determine if complementary mating types lacking *GFU2* form unresolved hemi-fusion membrane intermediates at the conjugation junction following pairing.

Unlike GFU2, GFU1 is clearly not a transmembrane protein, although its predicted structure resembles BAR domains on proteins that sense and contribute to membrane curvature^34,35^. This leaves open the possibility that GFU1 associates with the inner leaflet of the plasma membrane and contributes to membrane deformation necessary for pore initiation. Indeed, given its predicted structure and punctate pattern of staining, it would be tempting to speculate that GFU1 plays a role in the formation of hundreds of finger-like projections that emanate from membranes on either side at the conjugation junction and play a role in membrane fusion^24^. BAR-domain containing proteins are closely linked to the formation of filopodia, lamellipodia, and related membrane outpocketings in other systems^34,35,40,41^ and could have a similar role in *Tetrahymena* as well. As with the unitary *plus* and *minus* gamete mating structures of *C. reinhardtii*, membrane pore opening occurs at the distal tips of these finger-like projections in *T. thermophila*^24,25^ and one might predict that cells lacking proteins involved in the formation of these structures would behave similarly to *Δhap2* deletion strains. Consistent with that argument, *Δgfu1* deletion strains exhibit the same bilateral requirement for efficient membrane pore formation as *Δhap2* knockout lines. Furthermore, when crossed to each other, *Δgfu1* and *Δhap2* strains are completely unable to fuse (Fig. 2I) suggesting that their corresponding gene products may be functionally intertwined (see below). Lastly, BAR-domain-containing proteins have been implicated in cell-cell fusion events in mammals^42,43^, and at least one such protein, FUS7/RV161, has been shown to be required for fusion during mating of fission yeast^44,45^.

While additional work is needed to define the precise roles of GFU1 and GFU2, evidence of cooperativity between the fusion machinery on mating cells raises important questions regarding the mechanisms of HAP2-mediated gamete fusion, not just in *Tetrahymena* but in sexually dichotomous species such as *Arabidopsis thaliana* where the protein is only present on the surfaces of male gametes. In *Arabidopsis*, HAP2 drives gamete fusion from a single membrane. Nevertheless, in mammalian cells, syncytia formation occurs only when the plant ortholog is expressed on the membranes of adjacent cells^6^. This unusual bilateral requirement in a heterologous system may reflect an absence of key factors that otherwise facilitate gamete fusion in *A. thaliana*. Nevertheless, a bilateral requirement for cell-cell fusion has also been shown for the developmental fusogens AFF1 and EFF1 in *Caenorhabditis elegans*, both in vivo during worm development and following heterologous expression in tissue culture cells^46,47^. AFF1 and EFF1 are also class II fusion proteins, but while structurally homologous to HAP2, appear to lack hydrophobic fusion loops required to bridge adjacent membranes^48^. This has led to models in which trans-interactions between AFF1 or EFF1 protomers on adjacent cells result in membrane bridging, oligomerization, and fusion pore opening after conformational foldback of trimers formed from protomers on apposed cells^48–51^. Certainly, one could posit similar models for HAP2-dependent fusion in mating *Tetrahymena*, with the caveat that, unlike the nematode proteins, HAP2 has hydrophobic loops that are required for fusion^4,20^.

These and other considerations described below suggest alternative models that might explain cooperativity between the HAP2 fusion machinery in *T. thermophila*. The first is a hybrid of a previously proposed model described for AFF1 and EFF1^20,48,49^. It proposes direct interactions between the HAP2 ectodomains on mating cells and includes insertion of fusion loops into apposed membranes as a necessary step in generating fusion pores (Model 1, Fig. S5A). Such a model could explain the wild-type levels of fusion seen in crosses between *R*^*164*^*A* mutant and WT cells if the mutant protein (presumably lacking stable fusion loops) interacts with native protomers on the apposed membranes. Nevertheless, steric considerations around trimerization of proteins emanating from different membranes along with recent studies emphasizing the importance of hydrophobic fusion loops in formation of functional HAP2 trimers^9,10^ make the first model less appealing.

The second model, which we strongly favor, focuses on the membrane protrusions that extend across the luminal junction separating mating cells^24,25^ (Fig. S5B-D). As noted above, evidence for the involvement of such protrusions in HAP2-mediated fusion in *Tetrahymena* and *Chlamydomonas* suggests that trans-interactions between membranes at the tips of these structures (rather than between the HAP2 protomers, as in Model 1) are essential for the formation of fusion pores. In this context it is worth noting that actin-dependent invasive protrusions enhance EFF1-dependent syncytia formation in cultured insect cells, which appears to involve bilateral interactions as well^52^. Indeed, the involvement of microvilli and other membrane protrusions in cell-cell fusion^53,54^, as well as virus-host-cell fusion has long been argued^55,56^.

Based on these considerations, our second model (Model 2, Fig. 5) proposes that curved membranes at the tips of membrane protrusions destabilize the lipid bilayer locally creating an energetically favorable environment for fusion pore formation independent of HAP2-driven conformational foldback. In this model, HAP2 would play a dual role in helping to shape curvature at the tips of membrane protrusions while at the same time providing the motive force necessary to drive membrane merger (Fig. S5B,C). Efficient pore formation would require the presence of HAP2 in both membranes of a mating pair, not due to trans-interactions between protomers, but to the ability of the fusogen to contribute to curvature on interacting membranes (again, independent of its role in force generation).

Such a scenario would explain the low level of fusion seen in the absence of HAP2 on one membrane, as well as the wild-type levels of fusion seen in crosses between WT and *R*^*164*^*A* mutant strains (Fig. 4C). In WT × *Δhap2* crosses, the energy barrier to membrane merger would be difficult to overcome in the absence of curved membrane structures on the *Δhap2* deletion strain (Fig. S5D) leading to a reduced efficiency of pore formation. On the other hand, in *R*^*164*^*A* × WT crosses, so long as the mutant protein imparts membrane curvature in one cell and the native protein both curvature and functional fusion loops in the other, fusion pores would readily form (Fig. S5C). Based on this model, we propose that species in which HAP2 is expressed in only one gamete (*A. thaliana*, *C. reinhardtii*, etc.) would still require curved membrane protrusions on both cells of a mating pair but would resort to other proteins to shape these structures in the gamete lacking HAP2. It should also be noted that, while Model 2 posits tip-to-tip interactions, other possibilities exist including interactions of curved membrane projections with flatter regions of membrane on apposed cells that are nevertheless destabilized by HAP2.

Given its structural resemblance to BAR-domains, the potential involvement of GFU1 in this context should also be considered. In Model 2, GFU1 is shown contributing to curvature at the tips of membrane projections through its interaction with the inner leaflet of the lipid bilayer and, possibly, with HAP2 as well (Fig. S5C). In this scenario, GFU1 would be required in both cells as part of larger protein complexes that would include the fusogen itself (Fig. S5D).

Viral class II fusion proteins contribute to membrane curvature during virus assembly through protein-protein and possibly protein-lipid interactions^57^, and while we are unaware of evidence for the involvement of HAP2 in promoting curvature on membrane structures during fertilization, ultrastructural studies in *Tetrahymena* have yielded interesting observations along these lines. In crosses between *Δhap2* knockout strains, membrane protrusions are initiated at the conjugation junction, however, membrane pores fail to form. Instead, large blebs or vesicles appear in the luminal junction between cells that may be remnants of membrane outpocketings that failed to fuse^25^. If that interpretation is correct, the appearance of abnormal membrane structures in crosses between *Δhap2* deletion strains would support the idea that the fusogen itself plays a role in shaping the projections formed by WT cells.

Apart from mechanistic considerations, the expression of HAP2 on apposed membranes of mating cells has additional implications, both in evolutionary terms, and for fertilization success in organisms with multiple mating types, such as *Tetrahymena*. In the first instance, expression of HAP2 in all seven mating types of *T. thermophila* may simply reflect an early isogametic lifestyle in which the expression of “male” and “female” genes were not yet restricted to a given mating type^25^. If true (i.e., if cooperativity between the HAP2 fusion machinery on mating pairs represents the evolutionary ground state) its restricted expression in sexually dichotomous species would have required the emergence of other proteins that could compensate for its absence on the apposed membranes of partnering cells. On the second point, species with polygamous lifestyles and more than three mating types essentially require that gamete fusion proteins (in this case, HAP2) are expressed in all mating types if all pairwise matings are to succeed. In the case of *Tetrahymena*, mating type recognition and adherence is determined by proteins encoded at the mating type locus^58^. Nevertheless, even if cells of complementary mating types recognize and adhere to one another, fertilization will not occur unless at least one cell of any given pair expresses HAP2^4^, a requirement that would not be satisfied if any two mating types fail to express the protein.

Finally, while pair stability was closely correlated with membrane pore formation in strains lacking *GFU1*, *GFU2*, and *HAP2*, we were surprised that pairs formed by *Δzfr1* knockout lines were able to generate fusion pores despite being mechanically unstable. Nevertheless, based on the data shown in Fig. 5B, a hierarchy in pair stability appears to exist with WT × WT pairs being the most stable, followed by *Δzfr1* × *Δzfr1*, and then *Δhap2* × *Δhap2* pairs, which are completely unable to form pores^25^. Alterations to the number or size of fusion pores or the kinetics of pore opening could account for variations in pair stability as well as the low level of fertility seen in crosses between *Δzfr1* strains, particularly if the latter results from a failure of haploid pronuclei to migrate across the conjugation junction as hypothesized by Xu et al.^30^

Accordingly, *Δzfr1* × *Δzfr1* pairs may form fewer pores, smaller pores, or pores that close prematurely relative to WT × WT pairs. Indeed, given its localization^30^ and structural resemblance to E3 ubiquitin ligases (Fig. S4), it would not be surprising if ZFR1 regulates the half-lives of proteins at the conjugation junction including those involved in membrane dynamics. Certainly, ultrastructural studies examining the roles of ZFR1, GFU1, and GFU2 on membrane pore formation and expansion are warranted.

## Methods

### Growth and mating of *Tetrahymena thermophila* cell cultures.

Wild-type *T. thermophila* strains B2086; CU427; and CU428 were obtained from the Tetrahymena Stock Center (https://tetrahymena.vet.cornell.edu/). Cells of different mating type (MT) were grown separately to mid-log phase at 30°C with continuous shaking (100 rpm) in either NEFF medium (0.25% proteose peptone, 0.25% yeast extract, 0.5% glucose, 33.3 μM FeCl_3_) or SPP medium (2% proteose peptone, 0.1% yeast extract, 0.2% glucose, 33μM FeCl_3_)^59^ as indicated below. Prior to mating, cells were centrifuged at 400 × g for 2 min, washed in 10mM Tris buffer (pH 7.5), and then starved in the same buffer for 12–48 hr at 30°C. Following starvation, cells of different mating type were counted, brought to final concentrations of 2.5 × 10^5^ cells per mL and combined at ratios of 1:1 in 10 cm diameter plastic petri dishes. Mating cultures were then maintained without shaking at 30°C before being analyzed.

### Construction of *T. thermophila deletion strains*.

Different strategies were used to generate macronuclear gene disruptions of *GFU1, GFU2*, and *ZFR1*. For *GFU1*, the “co-deletion” method was used, which relies on the inherent genome editing properties of *T. thermophila* during macronuclear development^60^. Briefly, a 1562-bp target sequence covering roughly two thirds of the coding region between primers #1 and #2 (Table S2) in the predicted *GFU1* gene was cloned into a unique *Not*I site in the plasmid vector, pMcoDel^60^. The resulting construct contained the sequence targeted for deletion flanked by internally eliminated sequence motifs in a vector harboring the entire ribosomal DNA locus including a 17S rRNA allele which confers paromomycin resistance on transformed cells. Following vector construction, plasmid DNA was introduced into mating pairs of *T. thermophila* strains B2086 and CU428 at 7 hr post-mixing using biolistic bombardment as described by Bruns and Cassidy-Hanley^61^. Cells were maintained overnight in 10 mM Tris (pH7.5), grown for 3 hr in SPP medium, and drug-resistant positive transformants were selected by continuous growth in SPP containing 100 μg/mL paromomycin. A total of seven independent clones were identified, all of which showed the minimal 1562-bp deletion in the endogenous *GFU1* macronuclear DNA locus as determined by PCR using flanking primers. Complete elimination of the target sequence in all 45 macronuclear chromosomes was confirmed by quantitative PCR (qPCR) using primers #3 and #4 within the deleted region (Table S2). Three clones designated Loidl9 (MT = II), Loidl19 (MT = V), and Loidl21 (MT = IV) were used in these studies.

For disruption of *GFU2*, we used homologous recombination to replace the entire open reading frame of the gene with a cadmium-inducible cycloheximide (CHX) resistance cassette described by Gao et al^62^. In this case, regions containing >700-bp immediately upstream and downstream of the predicted *GFU2* coding sequence were amplified in separate PCR reactions using primers #5-#8 (Table S2) and linked on either side of the CHX cassette in pBlueScript SK(+) by Gibson assembly^63^. Plasmid DNA was then linearized and introduced into *T. thermophila* (B2086 X CU428) mating pairs by biolistic bombardment as above^61^. Positive transformants were selected by growth in SPP medium containing increasing concentrations (from 15 to 240 μg/mL) of cycloheximide and 4.5 μg/mL CdCl_2_^62,64^. Complete replacement of the endogenous *GFU2* gene by the CHX cassette was confirmed by qPCR using primers #9 and #10 (Table S2). Clonal isolates B3 (MT = II), C1 and C9 (both MT = VII) were used for all experiments.

For construction of *ZFR1* deletion strains, we used the “Neo4” paromomycin resistance cassette^65^ (available through the Tetrahymena Stock Center) to replace the entire macronuclear coding sequence of the gene by homologous recombination in *T. thermophila* strains containing different mating type backgrounds (CU427 [MT = VI] and CU428 [MT = VII]). A replacement vector containing the knockout construct was made by separately amplifying 5’- and 3’-flanking regions of the *ZFR1* gene via PCR using *T. thermophila* genomic DNA as the template and primer pairs ZFR1 5’Flankfor/ZFR1 5’flankrevXhoI and ZFR1 3’FLANKFORSacI/3’FLANK REV, respectively (Table S2). The resistance cassette was amplified from an existing Neo4 vector using the primer pair Neo4for2XhoI/Neo4cyrevSacI (Table S2). The three PCR products were then cut with restriction enzymes X*ho*I, S*ac*I, or both as appropriate, gel purified and sequentially ligated in reactions using T4 DNA ligase (New England Biolabs). The final ligation product was gel purified, blunt-end cloned into pCR4Blunt-TOPO, and sequenced. The resulting plasmid was linearized and introduced into the somatic macronucleus of *T. thermophila* using biolistic bombardment^61^. Positive transformants were then selected by serial passage of clones in NEFF medium containing increasing concentrations of paromomycin (up to 800μg/mL)^66^. Complete replacement of the endogenous *ZFR1* gene was verified by PCR amplification of genomic DNA using primers spanning the Neo4 insertion site (ZFRrescueF1 and ZFRrescuerev, Table S2). Deletion strains used in these studies showed only one PCR fragment of the size expected for the knockout construct and were designated *ZFR1KOinCU427cl.3* (MT = VI) and *ZFR1KOinCU428cl.51* (MT = VII).

### Construction of *T. thermophila* strains expressing mutant HAP2 and tagged GFU1 proteins.

Cell lines carrying the *T. thermophila HAP2 R164A* mutant allele were constructed as previously described^67^. Briefly, site-directed mutagenesis was carried out to alter the sequence of the wild-type *HAP2* cDNA using a Q5^®^ Site-Directed Mutagenesis Kit (New England Biolabs). The mutant cDNA was then amplified using primers N7_R64A_F and N7_R164A_R (Table S2) and directionally cloned between *Bam*HI and *Kpn*I restriction sites in a previously constructed replacement vector containing the pHrpl29-B cycloheximide resistance cassette in a pCR^™^Blunt-*TOPO*^®^ plasmid backbone (ThermoFisher). This placed the mutant allele between roughly 1,000-bp segments immediately upstream or downstream of the 5’- and 3’-flanking regions of the *T. thermophila* HAP2 gene, along with an HA-tag at the C-terminus of the HAP2 coding sequence. Vector DNA was then linearized and introduced into the *T. thermophila Δhap2* knockout strains *ΔHAP2–428* clone 5 (MT = VII) and *ΔHAP2–427* clone 6 (MT = VI) by biolistic bombardment^61^. Positive transformants were isolated by growth in NEFF medium containing increasing concentrations cycloheximide up to 50 μg/mL and their genomic DNA amplified and sequenced to establish the presence of the mutant allele^67^.

Construction of mCherry- and HA-tagged GFU1 expressing strains was carried out using a modified PCR-based C-terminal epitope tagging approach described by Kataoka et al.^68^. pBluescript^™^ plasmid DNA constructs containing codon-optimized sequences for mCherry or an HA-epitope tag immediately upstream of a *T. thermophila* BTU1–3’ termination sequence and neo4 resistance cassette^65^ were kindly provided by Dr. Kazufumi Mochizuki (IGH, CNRS-UM, Montpellier, FR). Plasmid DNA was then used to amplify the region between the 5’-ends of the coding sequences of the mCherry or HA-epitope tags and the 3’-end of the neo4 cassette in separate PCR reactions using primers BamHI-mCherry_Fw1 and HindIII-Neo4-RV2 (for mCherry) or BamHI-HA_FW1 and HindIII-Neo4-RV2 (for HA) (Table S2). *T. thermophila* genomic DNA was then used as a template to amplify a roughly 1,000-bp segment immediately upstream of the 3’-stop codon in the macronuclear *GFU1* gene in two separate PCR reactions using the same forward primer (5’FW) and either of two linker primers designated 5’RVmCherry and 5’RV-HA, respectively (Table S2). Lastly, a third segment extending 863-bp downstream of the 3’-end of the *GFU1* coding sequence was generated using the forward primer, 3’FW, and reverse primer, 3’-RV (Table S2) with *T. thermophila* genomic DNA as the template. The PCR products from each of the reactions were then stitched together by overlap PCR. After agarose gel electrophoresis, bands of the expected sizes of the full-length products were purified and amplified with the 5’RACE-Outer and 3’RACE-Outer primers (Table S2) to create the 5’-*GFU1-mCherry*-*BTU1*-*neoGFU1-*3*’* and 5’-*GFU1-HA*-*BTU1*-*neo*-*GFU1-*3*’* insertion cassettes. Insertion cassettes were then cloned into the plasmid vector pMiniT2 (New England Biolabs) and sequenced. Plasmid DNA was linearized by treatment with *Xho*1 and introduced into the endogenous *GFU1* locus of *T. thermophila* strains CU428.2 (MT VII) and B2086.2 (MT II) by homologous recombination following biolistic bombardment^61^. Positive transformant clones were selected by growth in NEFF medium containing increasing concentrations of paromomycin (up to 800μg/mL). The 5’-*GFU1-mCherry*-*BTU1*-*neo*-*GFU1-*3*’* insertion cassette was also used to replace the endogenous *GFU1* gene in a previously generated *T. thermophila* strain, HAP2GFP522 (MT = IV)^25^, containing an inducible C-terminal GFP-tagged version of HAP2 at the β-tubulin1 locus for co-localization studies.

All *Tetrahymena* constructs were frozen for long-term storage^59^ and are available through the Tetrahymena Stock Center (https://tetrahymena.vet.cornell.edu/).

### Pair Stability Assays.

For undisturbed cultures, cells of complementary mating type were grown separately in NEFF medium to mid-log phase, then washed and starved as indicated above. Between 0–3 hr post-mixing, aliquots were collected every 30 min by gentle pipetting, and combined with an equal volume of IC Fixation Buffer (eBioscience) in 1.5ml plastic microcentrifuge tubes. For time points after 3 hr, cells were fixed every hour until mating was complete (18–24 hr post-mixing). The percentage of cells that were paired at each time point was then determined by counting representative samples from each time point under a dissecting microscope as described below for mechanically agitated cells.

Strength of cell-cell pairing was determined as previously described^25^. Briefly, complementary mating types were grown and starved as above, then combined at ratios of 1:1. At 2 and 4 hr post-mixing, 3mL of mating culture was removed and placed into a 15 mL plastic conical tube. The tube was then lowered onto a shaking Vortex-Genie 2 laboratory mixer set to speed 4.5. Cells were then vortexed for cumulative periods of 0 sec, 2 sec, 5 sec, and additional 5 sec intervals up to 45 sec. At each time point, 50 μL aliquots were gently removed and fixed in an equal volume of IC Fixation Buffer. The percentage of cells paired at each time point was calculated by counting a representative sample from each of the fixed aliquots under a dissecting microscope. A total of 100 subjects (single cells or pairs) were counted for each time point with the percentage of pairs calculated as the number of paired cells divided by the number of total cells × 100.

### Flow Cytometric fusion assays.

Unless otherwise noted, the ability of mating pairs to form fusion pores was quantitated by flow cytometry as described by Pinello and co-workers^67^. Briefly, cells of complementary mating types were grown separately in NEFF medium to mid-log phase prior to starvation in Tris buffer (pH 7.5) at 30°C overnight. Starved cells were then centrifuged and resuspended in 0.1x phosphate buffered saline (PBS) to a final concentration of 7 × 10^6^ cells/mL. Aliquots of concentrated cells (1 mL) were combined with 1 mL 0.1x PBS containing either 2 μl Carboxyfluorescein diacetate succinimidyl ester (CFSE) (eBioscience) or 5 μl CellTrace^™^ Far Red (CTFR) (Invitrogen^™^) and incubated 5 min (CFSE) or 15 min (CTFR) before quenching unincorporated dye in 10 mL of NEFF medium. Labeled ells were pelleted at 400 × g, then washed twice and resuspended in 10 mM Tris·HCl buffer (pH 7.5) at 30°C in the dark. The next day, cells were resuspended in fresh 10 mM Tris·HCl buffer (pH 7.5) and recounted. Aliquots of 5 × 10^5^ cells of each mating type were then combined in a total of 5 mL of 10 mM Tris buffer in triplicate plates, and either collected immediately or allowed to mate at 30°C for 18–36 hr (as indicated in the text) in a darkened incubator. Following mating, cells were harvested by centrifugation at 400 × g, resuspended in 1 mL of 10 mM Tris buffer and combined with 1 mL of IC Fixation Buffer followed by incubation in the dark for a minimum of 20 min. After fixation, cells were again harvested and resuspended in 500 μL of 1x PBS containing 0.3% bovine serum albumin (Sigma-Aldrich) prior to acquisition on a FACSCanto^™^ II flow cytometer (Becton Dickinson) using the FITC-A and APC-CY7-A lasers. A minimum of 30,000 events were acquired for each sample. Data were analyzed using FlowJo version 10.4 software with gates of single-(unfused), double-labeled (fused) and paired cell populations being drawn as previously described^67^. Compiled data from all experiments for a given cross were assembled as bar charts using Prism Software (GraphPad, Inc.).

### Syncytia formation assays in mammalian cell culture.

Full-length, codon-optimized cDNA sequences encoding *A. thaliana* HAP2 and *T. thermophila* GFU1 and GFU2 were introduced into a pCAGGS plasmid expression vector (TWIST Bioscience) downstream of a cytomegalovirus early enhancer and chicken β-actin promoter and in-frame with the C-terminal tags Au1 (*A. thaliana* HAP2); HA (GFU1); or FLAG (GFU2). For *T. thermophila* HAP2/GCS1, a full-length, codon-optimized version of the corresponding cDNA containing a C-terminal 3X-FLAG tag was cloned into the plasmid expression vector pcDNA3.1(+) as previously described by Pinello et al.^67^ For syncytia assays, BSR-T7 cells (gift from Ben Benhur Lee, Icahn School of Medicine at Mount Sinai) were seeded onto 6-well plates (VWR, 734–2777) and grown in 1x DMEM medium (Corning, 10–013-CV) containing 10% bovine calf serum and 1% penicillin/streptomycin. When monolayers reached 80% confluence, cells were transfected with plasmid DNA using Lipofectamine 2000 Transfection Reagent^™^ according to the manufacturer’s instructions (Invitrogen^™^, 11668–019). Constant concentrations of plasmid DNA across conditions were maintained by combining 1 μg HAP2encoding plasmid DNA with either empty vector (2 μg) or 1 μg each of vector DNA encoding *T. thermophila* GFU1 and GFU2. Expression of epitope-tagged proteins was verified 24 hr post-transfection by western blotting and fluorescence microscopy using labeled antibodies against specific tags in each case. Expression levels of the *Arabidopsis* and *Tetrahymena* HAP2 proteins were roughly equivalent based on densitometry readings in western blots. To assay syncytia formation, cultures were fixed in 1x PBS (pH 7.4) containing 2% paraformaldehyde at 30 hr post-transfection and stained with Hoechst 33342 (Thermo Scientific, 62249) to label nuclei. Syncytia formation was quantified by measuring the total number of nuclei in syncytia per field in 4–7 random fields per experiment (n=3) using an inverted microscope (ECHO Revolve) with phase optics at 10x magnification. A syncytium was considered any cell with ≥4 nuclei.

### Fluorescence Microscopy.

For visualization of HA-tagged GFU1, mating *T. thermophila* cells were centrifuged at 400 × g, resuspended in 20 mM HEPES buffer (pH 7.5) and fixed with IC Fixation Buffer as described above for flow cytometry. Cells were then resuspended and washed three times in 1 mL of 1x Permeabilization Buffer in a 1.5 mL plastic microcentrifuge tube before being resuspended in blocking solution (1x Permeabilization buffer containing 5% Goat Serum and 1% BSA). The same blocking solution was used for all subsequent washes and antibody incubations. After blocking for a minimum of 30 min at room temperature, cells were again washed and then incubated at 4°C overnight in blocking solution containing a 1:100 dilution (100 μg/mL) of primary antibody (Anti-HA, clone 3F10) (Catalog # 50–100-3325; Fisher Scientific). Following incubation, cells were washed 3X in blocking solution before being incubated in the dark at RT with a 1:400 dilution (10 μg/mL) of FITC-conjugated secondary goat Anti-Rat IgG (H+L) (Catalog # 401414; Sigma-Aldrich). Following incubation, cells were washed 3 times and resuspended in a small volume of blocking solution for imaging on a Leica SP5 confocal microscope.

For co-localization studies, the HAP2GFP522 strain described above containing an mCherry-tagged version of *GFU1* at the endogenous locus and an inducible GFP-tagged version of HAP2 at the b-tubulin locus were induced with 0.1 μg/mL CdCl_2_, 2 hr prior to mating. Following induction, transgenic cells were mixed at ratios of 1:1 with wild-type strain CU428.2. At varying times thereafter, mating pairs were examined with an Olympus BX-50 fluorescence microscope equipped with a Fluoview scanning laser confocal imaging system and images captured with a DP-72 digital camera.

### Protein structure modeling.

The 3D structure prediction of GFU1, GFU2, and ZFR1 were done with a local installation Alphafold^33^ version 2.3.1 at the Institut Pasteur, using the latest release of the protein databank available in March 2023. Only predictions with highest scores are displayed in the Figures. Additionally, Phyre2^69^ and RaptorX^70^ algorithms were used to model the structures of ZFR1 and GFU1, respectively. For Phyre2, we used the Protein Fold Recognition Server at http://www.sbg.bio.ic.ac.uk/~phyre2/html/page.cgi?id=index. For RaptorX, we used the server http://raptorx6.uchicago.edu/. Details of p-value, score, uGDT/GDT, and uSeqID/SeqID in Fig. 3C are provided at http://raptorx6.uchicago.edu/documentation/.

### Fertilization Assays.

Mating success in crosses between *Δzfr1* deletion strains was determined by assaying progeny cells for resistance to growth in either cycloheximide (CY), 6-methlypuriine (6MP), or both drugs after mating. As indicated above (**Construction of *T. thermophila* deletion strains**), *ZFR1* was deleted in the parental cell lines CU427 and CU428 with complementary mating type backgrounds (VI and VII) and carrying either a cycloheximide resistance (*Cyr*) or a 6-methylpurine resistance (*Mpr*) allele in their germline micronuclei, respectively. These deletion strains are wild-type in their transcriptionally active macronuclei and are sensitive to either drug. To determine mating success in crosses between deletion strains, cells were grown to mid-log phase, starved in 10 mM Tris buffer (pH 7.4) and mated at a ratio of 1:1. At 6–9.5 hr post-mixing, individual pairs were hand-isolated into hanging drops to establish “synclones,” or mixed populations of the karyonidal descendants from each exconjugant. Following transfer to 96-well plates, synclones were subjected to sequential drug testing in 25 μg/mL cycloheximide followed by 25 μg/mL 6-methylpurine and examined for survival 36–48 hr after drug treatment using a dissecting microscope. True “cross-fertilized” progeny are synclones that were resistant to both drugs; “self-fertilizers” or “cytogamonts” were resistant to either CY or 6MP; while “backouts” (parental cells that failed to successfully mate) were sensitive to both CY and 6MP.

## References

[1] Mori T., Kuroiwa H., Higashiyama T. & Kuroiwa T. GENERATIVE CELL SPECIFIC 1 is essential for angiosperm fertilization. Nat Cell Biol 8, 64–71 (2006). 16378100 10.1038/ncb1345

[2] Wong J. L. & Johnson M. A. Is HAP2-GCS1 an ancestral gamete fusogen? Trends in Cell Biology 20, 134–141 (2010). 20080406 10.1016/j.tcb.2009.12.007

[3] Speijer D., Lukeš J. & Eliáš M. Sex is a ubiquitous, ancient, and inherent attribute of eukaryotic life. Proc Natl Acad Sci U S A 112, 8827–8834 (2015). 26195746 10.1073/pnas.1501725112 PMC4517231

[4] Pinello J. F. Structure-Function Studies Link Class II Viral Fusogens with the Ancestral Gamete Fusion Protein HAP2. Curr Biol 27, 651–660 (2017). 28238660 10.1016/j.cub.2017.01.049 PMC5393271

[5] Fédry J. The Ancient Gamete Fusogen HAP2 Is a Eukaryotic Class II Fusion Protein. Cell 168, 904–915.e10 (2017). 28235200 10.1016/j.cell.2017.01.024 PMC5332557

[6] Valansi C. Arabidopsis HAP2/GCS1 is a gamete fusion protein homologous to somatic and viral fusogens. Journal of Cell Biology 216, 571–581 (2017). 28137780 10.1083/jcb.201610093 PMC5350521

[7] Baquero E., Fedry J., Legrand P., Krey T. & Rey F. A. Species-Specific Functional Regions of the Green Alga Gamete Fusion Protein HAP2 Revealed by Structural Studies. Structure 27, 113–124.e4 (2019). 30416037 10.1016/j.str.2018.09.014 PMC6327110

[8] Fedry J. Evolutionary diversification of the HAP2 membrane insertion motifs to drive gamete fusion across eukaryotes. PLoS Biol 16, e2006357 (2018). 30102690 10.1371/journal.pbio.2006357 PMC6089408

[9] Zhang J. Species-specific gamete recognition initiates fusion-driving trimer formation by conserved fusogen HAP2. Nat Commun 12, 4380 (2021). 34282138 10.1038/s41467-021-24613-8 PMC8289870

[10] Feng J., Dong X., Su Y., Lu C. & Springer T. A. Monomeric prefusion structure of an extremophile gamete fusogen and stepwise formation of the postfusion trimeric state. Nat Commun 13, 4064 (2022). 35831325 10.1038/s41467-022-31744-z PMC9279424

[11] Angrisano F. Targeting the Conserved Fusion Loop of HAP2 Inhibits the Transmission of Plasmodium berghei and falciparum. Cell Reports 21, 2868–2878 (2017). 29212032 10.1016/j.celrep.2017.11.024 PMC5732318

[12] Feng J. Fusion surface structure, function, and dynamics of gamete fusogen HAP2. eLife 7, e39772 (2018). 30281023 10.7554/eLife.39772 PMC6170185

[13] Kielian M. Mechanisms of Virus Membrane Fusion Proteins. Annu. Rev. Virol. 1, 171–189 (2014). 26958720 10.1146/annurev-virology-031413-085521

[14] Guardado-Calvo P. & Rey F. A. The Viral Class II Membrane Fusion Machinery: Divergent Evolution from an Ancestral Heterodimer. Viruses 13, 2368 (2021). 34960636 10.3390/v13122368 PMC8706100

[15] Leikina E. Myomaker and Myomerger Work Independently to Control Distinct Steps of Membrane Remodeling during Myoblast Fusion. Developmental Cell 46, 767–780.e7 (2018). 30197239 10.1016/j.devcel.2018.08.006 PMC6203449

[16] Pinello J. F., Liu Y. & Snell W. J. MAR1 links membrane adhesion to membrane merger during cell-cell fusion in Chlamydomonas. Developmental Cell S1534580721008546 (2021) doi:10.1016/j.devcel.2021.10.023. PMC8794015 34813735

[17] Liu Y. The conserved plant sterility gene HAP2 functions after attachment of fusogenic membranes in Chlamydomonas and Plasmodium gametes. Genes & development 22, 1051–1068 (2008). 18367645 10.1101/gad.1656508 PMC2335326

[18] Wang W. DMP8 and 9 regulate HAP2/GCS1 trafficking for the timely acquisition of sperm fusion competence. Proc. Natl. Acad. Sci. U.S.A. 119, e2207608119 (2022). 36322734 10.1073/pnas.2207608119 PMC9659367

[19] Sprunck S. Egg cell-secreted EC1 triggers sperm cell activation during double fertilization. Science (New York, N.Y.) 338, 1093–1097 (2012). 23180860 10.1126/science.1223944

[20] Pinello J. F. & Clark T. G. HAP2-Mediated Gamete Fusion: Lessons From the World of Unicellular Eukaryotes. Front. Cell Dev. Biol. 9, 807313 (2022). 35071241 10.3389/fcell.2021.807313 PMC8777248

[21] Cole E. & Sugai T. Developmental progression of Tetrahymena through the cell cycle and conjugation. Methods in cell biology 109, 177–236 (2012). 22444146 10.1016/B978-0-12-385967-9.00007-4

[22] Cervantes M. D. Selecting one of several mating types through gene segment joining and deletion in Tetrahymena thermophila. PLoS Biol 11, e1001518 (2013). 23555191 10.1371/journal.pbio.1001518 PMC3608545

[23] Cole E. S. The Tetrahymena Conjugation Junction. in Cell-Cell Channels 39–62 (Springer New York, 2006). doi:10.1007/978-0-387-46957-7_3.

[24] Cole E. S. Membrane dynamics at the nuclear exchange junction during early mating (one to four hours) in the ciliate Tetrahymena thermophila. Eukaryot Cell 14, 116–127 (2015). 25107923 10.1128/EC.00164-14 PMC4311919

[25] Cole E. S. Function of the male-gamete-specific fusion protein HAP2 in a seven-sexed ciliate. Current biology : CB 24, 2168–2173 (2014). 25155508 10.1016/j.cub.2014.07.064 PMC4313389

[26] Orias J. D., Hamilton E. P. & Orias E. A microtubule meshwork associated with gametic pronucleus transfer across a cell-cell junction. Science 222, 181–184 (1983). 6623070 10.1126/science.6623070

[27] Steele R. E. & Dana C. E. Evolutionary History of the HAP2/GCS1 Gene and Sexual Reproduction in Metazoans. PLoS ONE 4, e7680 (2009). 19888453 10.1371/journal.pone.0007680 PMC2766252

[28] Mori T., Kuroiwa H., Higashiyama T. & Kuroiwa T. GENERATIVE CELL SPECIFIC 1 is essential for angiosperm fertilization. Nature cell biology 8, 64–71 (2006). 16378100 10.1038/ncb1345

[29] Orias E. Membrane fusion: HAP2 protein on a short leash. Curr Biol 24, R831–R833 (2014). 25247352 10.1016/j.cub.2014.08.004

[30] Xu J., Tian H., Wang W. & Liang A. The zinc finger protein Zfr1p is localized specifically to conjugation junction and required for sexual development in Tetrahymena thermophila. PLoS One 7, e52799 (2012). 23251712 10.1371/journal.pone.0052799 PMC3519685

[31] Xiong J. Tetrahymena functional genomics database (TetraFGD): an integrated resource for Tetrahymena functional genomics. Database (Oxford) 2013, bat008 (2013). 10.1093/database/bat008 PMC3594985 23482072

[32] Loidl J. Tetrahymena meiosis: Simple yet ingenious. PLoS Genet 17, e1009627 (2021). 34264933 10.1371/journal.pgen.1009627 PMC8282021

[33] Jumper J. Highly accurate protein structure prediction with AlphaFold. Nature 596, 583–589 (2021). 34265844 10.1038/s41586-021-03819-2 PMC8371605

[34] Simunovic M., Evergren E., Callan-Jones A. & Bassereau P. Curving Cells Inside and Out: Roles of BAR Domain Proteins in Membrane Shaping and Its Cellular Implications. Annu. Rev. Cell Dev. Biol. 35, 111–129 (2019). 31340125 10.1146/annurev-cellbio-100617-060558

[35] Peter B. J. BAR domains as sensors of membrane curvature: the amphiphysin BAR structure. Science 303, 495–499 (2004). 14645856 10.1126/science.1092586

[36] Contreras E. M. Roles of Cholesterol in Early and Late Steps of the Nipah Virus Membrane Fusion Cascade. J Virol 95, e02323–20 (2021). 33408170 10.1128/JVI.02323-20 PMC8094960

[37] Cyprys P., Lindemeier M. & Sprunck S. Gamete fusion is facilitated by two sperm cellexpressed DUF679 membrane proteins. Nat. Plants 5, 253–257 (2019). 30850817 10.1038/s41477-019-0382-3

[38] Takahashi T. The male gamete membrane protein DMP9/DAU2 is required for double fertilization in flowering plants. Development 145, dev170076 (2018). 10.1242/dev.170076 30487178

[39] Chen B., You W., Wang Y. & Shan T. The regulatory role of Myomaker and Myomixer–Myomerger–Minion in muscle development and regeneration. Cell. Mol. Life Sci. 77, 1551–1569 (2020). 31642939 10.1007/s00018-019-03341-9 PMC11105057

[40] Zhao H., Pykäläinen A. & Lappalainen P. I-BAR domain proteins: linking actin and plasma membrane dynamics. Current Opinion in Cell Biology 23, 14–21 (2011). 21093245 10.1016/j.ceb.2010.10.005

[41] Fox S., Tran A., Trinkle-Mulcahy L. & Copeland J. W. Cooperative assembly of filopodia by the formin FMNL2 and I-BAR domain protein IRTKS. Journal of Biological Chemistry 298, 102512 (2022). 36259517 10.1016/j.jbc.2022.102512 PMC9579038

[42] Doherty J. T. Skeletal Muscle Differentiation and Fusion Are Regulated by the BAR-containing Rho-GTPase-activating Protein (Rho-GAP), GRAF1. Journal of Biological Chemistry 286, 25903–25921 (2011). 21622574 10.1074/jbc.M111.243030 PMC3138304

[43] Richard J.-P. Intracellular curvature-generating proteins in cell-to-cell fusion. Biochemical Journal 440, 185–193 (2011). 21895608 10.1042/BJ20111243 PMC3216009

[44] Ren G., Vajjhala P., Lee J. S., Winsor B. & Munn A. L. The BAR Domain Proteins: Molding Membranes in Fission, Fusion, and Phagy. Microbiol Mol Biol Rev 70, 37–120 (2006). 16524918 10.1128/MMBR.70.1.37-120.2006 PMC1393252

[45] Brizzio V., Gammie A. E. & Rose M. D. Rvs161p Interacts with Fus2p to Promote Cell Fusion in Saccharomyces cerevisiae. Journal of Cell Biology 141, 567–584 (1998). 9566960 10.1083/jcb.141.3.567 PMC2132759

[46] Podbilewicz B. The C. elegans Developmental Fusogen EFF-1 Mediates Homotypic Fusion in Heterologous Cells and In Vivo. Developmental Cell 11, 471–481 (2006). 17011487 10.1016/j.devcel.2006.09.004

[47] Sapir A. AFF-1, a FOS-1-Regulated Fusogen, Mediates Fusion of the Anchor Cell in C. elegans. Developmental Cell 12, 683–698 (2007). 17488621 10.1016/j.devcel.2007.03.003 PMC1975806

[48] Perez-Vargas J. Structural basis of eukaryotic cell-cell fusion. Cell 157, 407–419 (2014). 24725407 10.1016/j.cell.2014.02.020

[49] Zeev-Ben-Mordehai T., Vasishtan D., Siebert C. A. & Grünewald K. The full-length cell–cell fusogen EFF-1 is monomeric and upright on the membrane. Nat Commun 5, 3912 (2014). 24867324 10.1038/ncomms4912 PMC4050280

[50] Brukman N. G., Li X. & Podbilewicz B. Fusexins, HAP2/GCS1 and Evolution of Gamete Fusion. Front. Cell Dev. Biol. 9, 824024 (2022). 35083224 10.3389/fcell.2021.824024 PMC8784728

[51] Hernández J. M. & Podbilewicz B. The hallmarks of cell-cell fusion. Development 144, 4481–4495 (2017). 29254991 10.1242/dev.155523

[52] Shilagardi K. Actin-Propelled Invasive Membrane Protrusions Promote Fusogenic Protein Engagement During Cell-Cell Fusion. Science 340, 359–363 (2013). 23470732 10.1126/science.1234781 PMC3631436

[53] Faust J. J. An actin-based protrusion originating from a podosome-enriched region initiates macrophage fusion. MBoC 30, 2254–2267 (2019). 31242090 10.1091/mbc.E19-01-0009 PMC6743464

[54] Luo Z. The cellular architecture and molecular determinants of the zebrafish fusogenic synapse. Developmental Cell 57, 1582–1597.e6 (2022). 35709765 10.1016/j.devcel.2022.05.016 PMC10180866

[55] Wilson N. F. & Snell W. J. Microvilli and cell-cell fusion during fertilization. Trends in Cell Biology 8, 93–96 (1998). 9695816 10.1016/s0962-8924(98)01234-3

[56] Chen E. H. Invasive Podosomes and Myoblast Fusion. in Current Topics in Membranes vol. 68 235–258 (Elsevier, 2011). 21771502 10.1016/B978-0-12-385891-7.00010-6 PMC4373657

[57] Zhang W., Kaufmann B., Chipman P. R., Kuhn R. J. & Rossmann M. G. Membrane curvature in flaviviruses. Journal of Structural Biology 183, 86–94 (2013). 23602814 10.1016/j.jsb.2013.04.005 PMC4091808

[58] Cervantes M. D. Selecting one of several mating types through gene segment joining and deletion in Tetrahymena thermophila. PLoS Biol 11, e1001518 (2013). 23555191 10.1371/journal.pbio.1001518 PMC3608545

[59] Cassidy-Hanley D. M. Tetrahymena in the Laboratory: Strain Resources, Methods for Culture, Maintenance, and Storage. in Methods in Cell Biology vol. 109 237–276 (Elsevier, 2012). 22444147 10.1016/B978-0-12-385967-9.00008-6 PMC3608402

[60] Hayashi A. & Mochizuki K. Targeted Gene Disruption by Ectopic Induction of DNA Elimination in *Tetrahymena*. Genetics 201, 55–64 (2015). 26205990 10.1534/genetics.115.178525 PMC4566276

[61] Bruns P. J. & Cassidy-Hanley D. Chapter 27 Biolistic Transformation of Macro- and Micronuclei. in Methods in Cell Biology vol. 62 501–512 (Elsevier, 1999). 10.1016/s0091-679x(08)61553-8 10503214

[62] Gao S. Impaired replication elongation in *Tetrahymena* mutants deficient in histone H3 Lys 27 monomethylation. Genes Dev. 27, 1662–1679 (2013). 23884606 10.1101/gad.218966.113 PMC3744725

[63] Gibson D. G. Enzymatic assembly of DNA molecules up to several hundred kilobases. Nat Methods 6, 343–345 (2009). 19363495 10.1038/nmeth.1318

[64] Tian M. & Loidl J. A chromatin-associated protein required for inducing and limiting meiotic DNA double-strand break formation. Nucleic Acids Research (2018) doi:10.1093/nar/gky968. PMC6294514 30357385

[65] Mochizuki K. High efficiency transformation of Tetrahymena using a codon-optimized neomycin resistance gene. Gene 425, 79–83 (2008). 18775482 10.1016/j.gene.2008.08.007

[66] Pinello J. F. HAP2-MEDIATED CELL FUSION IN A SEXUAL CILIATE. (2017) doi:10.7298/X4N58JB1.

[67] Pinello J. F. Structure-Function Studies Link Class II Viral Fusogens with the Ancestral Gamete Fusion Protein HAP2. Current Biology 27, 651–660 (2017). 28238660 10.1016/j.cub.2017.01.049 PMC5393271

[68] Kataoka K., Schoeberl U. E. & Mochizuki K. Modules for C-terminal epitope tagging of Tetrahymena genes. Journal of Microbiological Methods 82, 342–346 (2010). 20624430 10.1016/j.mimet.2010.07.009 PMC2935961

[69] Kelley L. A., Mezulis S., Yates C. M., Wass M. N. & Sternberg M. J. E. The Phyre2 web portal for protein modeling, prediction and analysis. Nat Protoc 10, 845–858 (2015). 25950237 10.1038/nprot.2015.053 PMC5298202

[70] Wang S., Li W., Liu S. & Xu J. RaptorX-Property: a web server for protein structure property prediction. Nucleic Acids Res 44, W430–W435 (2016). 27112573 10.1093/nar/gkw306 PMC4987890

